# Supplementary material for: The Autophagy Protein Pacer Positively Regulates the Therapeutic Potential of Mesenchymal Stem Cells in a Mouse Model of DSS-Induced Colitis
Source: Cells. 2022 Apr 30;11(9):1503. doi: 10.3390/cells11091503 (PMC9101276; doi:10.3390/cells11091503)
Supplement: Supplementary file 1 [file cells-11-01503-s001.zip › cells-1677181-supplementary.pdf]

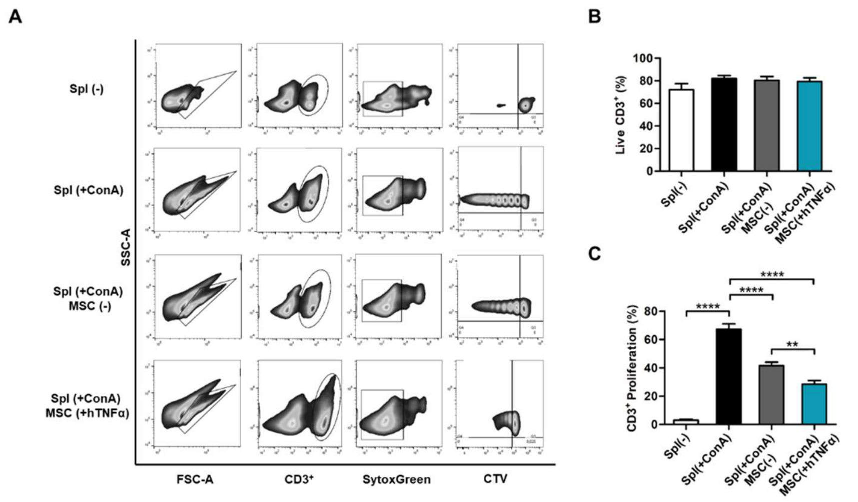

Figure S1. TNFα pre-treated MSC show augmented capacity to supress T-cell proliferation. (**A-C**) T-cell proliferation was evaluated by flow cytometry, gating on CD3+ cells as described in material and methods. Splenocytes were stimulated with ConA in absence or presence of MSC pre-treated or not with TNFα. (**A**) Live CD3+ cells were determined as SytoxGreen-negative stainning. CD3+ proliferation was assessed using Cell Trace Violet (CTV) staining. (**B**) Percentage of live CD3+ cells. (**C**) CD3+ cell proliferation. In (**B**) and (**C**) Mean ± SEM are shown. For statistical analysis one-way ANOVA with Tukey post-hoc test was performed. Only significant p values are shown. p values: *, p ≤ 0.05; **, p ≤ 0.01; ***, p ≤ 0.001; ****, p ≤ 0.0001.

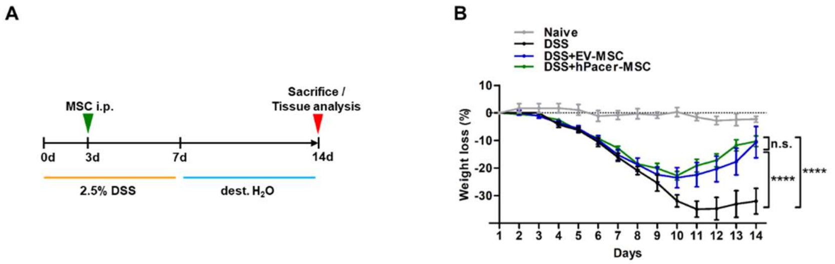

Figure S2. Experimental colitis and MSC treatment scheme. (**A**) Experimental scheme outlining the water schedule and duration of DSS treatment and time point of intraperitoneal (i.p.) MSC injection. (**B**) The weight loss percentage of naïve (n = 6), DSS (n = 7), DSS+EV-MSC (n = 8) and DSS+hPacer-MSC (n = 8) was determined daily. For statistical analysis one-way ANOVA with Tukey post-hoc test was performed. p values: ****, p ≤ 0.0001; n.s., non-significant.
